# Hospice care in the Netherlands: who applies and who is admitted to inpatient care?

**DOI:** 10.1186/s12913-016-1273-1

**Published:** 2016-01-28

**Authors:** Emily West, H. Roeline Pasman, Cilia Galesloot, Martine Elizabeth Lokker, Bregje Onwuteaka-Philipsen, On behalf of EURO IMPACT

**Affiliations:** 1 Department of Public and Occupational Health, EMGO Institute for Health and Care Research – Expertise Centre for Palliative Care, VU University medical center, Amsterdam, The Netherlands; 2 Department of Registry & Research, Comprehensive Cancer Centre the Netherlands (IKNL), PO Box 19079, 3501 DB Utrecht, The Netherlands

**Keywords:** Inpatient hospice care, Cancer, End of life, Palliative care, Nurses, Nursing

## Abstract

**Background:**

Ten percent of non-sudden deaths in the Netherlands occur in inpatient hospice facilities. To investigate differences between patients who are admitted to inpatient hospice care or not following application, how diagnoses compare to the national population, characteristics of application, and associations with being admitted to inpatient hospice care or not.

**Methods:**

Data from a database representing over 25 % of inpatient hospice facilities in the Netherlands were analysed. The study period spanned the years 2007–2012. Multivariate regression analyses were performed to study associations between demographic and application characteristics, and admittance.

**Results:**

Ten thousand two hundred fifty-four patients were included. 84.1 % of patients applying for inpatient hospice care had cancer compared to 37.0 % of deaths nationally. 52.4 % of applicants resided in hospital at the time of admission. Most frequent reasons for application were the wish to die in an inpatient hospice facility (70.5 %), needing intensive care or support (52.2 %), relieving caregivers (41.4 %) and needing pain/symptom control (39.9 %). Living alone (OR 1.68, 95 % CI 1.46–1.94), having cancer (OR 1.40, 95 % CI 1.11–1.76), relieving caregivers (OR 1.18, 95 % CI 1.01–1.38), needing pain/symptom control (OR1.72, 95 % CI 1.46–2.03) wanting inpatient hospice care until death (vs respite care) (OR 3.59, 95 % CI 2.11–6.10), wanting to be admitted as soon as possible (OR 1.64, 95 % CI 1.42–1.88), and being referred by a primary care professional (OR 1.36, 95 % CI 1.17–1.59) were positively associated with being admitted. Wishing to die in an inpatient hospice facility was negatively associated with being admitted (OR 0.85, 95 % CI 0.72–1.00).

**Conclusions:**

This study suggests that when applying for inpatient hospice care, patients who seem most urgently in need of inpatient hospice care are more frequently admitted. However, non-cancer patients seem to be an under-represented population. Staff should consider application based on need for palliation, irrespective of diagnosis.

## Background

End of life care exists in many forms in healthcare systems, from services fully integrated into acute healthcare settings to standalone units and peripatetic visiting teams. Hospice as a model of end-of-life care can take a number of different forms according to organisational strategies and the needs of patients. Inpatient hospice facilities in the Netherlands are organised into three main types, providing different types of caregiver support and integration with other models of care. The 2013 European Atlas of Palliative Care recorded 212 inpatient hospice facilities in the Netherlands, of which 55 were standalone hospice facilities, and 157 were palliative care units (PCUs) embedded in tertiary and non-tertiary care facilities, generally nursing homes [1]. Standalone hospice facilities are further divided into two types – inpatient ‘hospices’, which are staffed by nurses and physicians who are part of the institution and bijna-thuis-huizen (almost-home-homes) that have visiting nurses and physicians but the majority of care carried out by volunteers and families [2].

Currently 10 % of all non-sudden deaths in the Netherlands occur in inpatient hospice facilities [3]. The number of inpatient hospice facilities is growing rapidly; from 86 inpatient hospice facilities that were recorded in the 2007 edition of the EAPC European Atlas of Palliative care [4], to 212 in the 2013 edition [1]. Hospice as an institution was originally developed for cancer patients [5] and use of inpatient hospice facilities today still reflects this bias. European data have shown an imbalance in inpatient hospice use for certain groups when compared with the national average, with cancer patients consistently being found to be more likely to receive specialist palliative care (including in inpatient hospice facilities) than other groups such as patients with lung disease or heart failure [6]. Demographic differences have also been found to have an influence, with the oldest-old being less likely to receive specialist care at the end of life [7]. This unequal distribution of the use of inpatient hospice facilities may be related to different levels of need, but may also represent unequal levels of access [8].

This paper aims to investigate the differences between the populations who are admitted to inpatient hospice facilities or not following application, and how the spread of diagnoses compares to the national population at the end of life in terms of the characteristics of people who apply for care in inpatient hospice facilities. We will also investigate the characteristics of application in terms of what type of inpatient hospice facility is being applied for, where the patient is admitted from, the reason for application, the length of desired care, how urgent admission is, the person who referred to inpatient hospice care, when care in a hospice is sought and which patient and application characteristics are associated with being admitted to inpatient hospice facilities or not.

## Methods

This study utilised a Dutch database of patients applying for inpatient hospice care between 2007 and 2012 registered for the Netherlands hospice – database managed by the comprehensive cancer center of the Netherlands (IKNL). Data were gathered from 64 institutions that provide inpatient hospice care. Not all institutions provided data for all years, but provided data for one or more years of this timespan. Given the 212 inpatient hospice facilities in the Netherlands in 2013 [1], this means that around a quarter of all inpatient hospice facilities are involved in providing data.

The original database contained 10,502 cases. 248 records were removed, of which the majority were duplicate records. In addition, if a patient was recorded as having more than one admission, only the data from the final admission were used for analysis and other visits were removed from the database – this was the case for 21 patients, with number of visits varying between two and nine. In total 10,254 cases were included for analysis.

### Ethics

In accordance with Dutch law, this study was exempt from seeking approval from an ethical review board as there were no imposing actions or interventions involved. Data were de-identified, so that individual patients could not be recognised.

### Instrument

Applications to inpatient hospice care in the Netherlands are not centrally regulated or coordinated, and are made directly to individual hospices. Any person, including the patient and their informal caregivers, can make an application for inpatient hospice care. According to insurance regulations, inpatient hospice care is for patients in the final 3 months of life [9]. Healthcare professionals in participating institutions registered data using standardised electronic forms. The standardised form recorded demographic and application characteristics of patients before admittance, and characteristics of care received if the patient was admitted to inpatient hospice care.

Demographic variables included for analysis were age, gender, whether the patient lived alone or not and their primary diagnosis. Application variables were the type of inpatient hospice facility applied to, where the patient resided at the time of application, the stated reason for application, whether the patient wanted care until death or respite care, the desired timing for admission and who referred the patient to inpatient hospice care.

### Analysis

The study population was dichotomised into those who were admitted to inpatient hospice care, and those who applied but were not admitted. Differences between the two groups for demographic and application characteristics were assessed using chi-square testing.

To compare the spread of diagnoses for patients applying for inpatient hospice care with the main diagnosis of all people in the Netherlands who died non-suddenly, data on cause of death from a national death certificate study were used [10]. Non-sudden deaths were classified as all deaths that were not the result of an accident or sudden acute medical condition such as a stroke or cardiovascular accident.

To study the association between demographic and application characteristics and admittance to inpatient hospice care, we performed uni- and multivariate logistic regression analyses with being admitted to a hospice or not as dependent variable and demographic and application characteristics as independent variables. Analyses were firstly performed univariately, and independent variables that were significant were then entered backwards stepwise in multivariate analyses.

## Results

### Demographic patient characteristics

**Table 1:** Demographic Characteristics of Hospice Applicants

	All applicants (*n* = 10254)		Admitted to inpatient hospice facility (*n* = 7966)		Not admitted to inpatient hospice facility (*n* = 2288)		*P* value
Age	n	%	n	%	n	%	
19–40	74	0.8	52	0.7	22	1.0	
41–60	1182	12.3	930	12.4	252	12.0	
61–80	4665	48.6	3678	49.0	987	47.0	
81+	3682	38.3	2845	37.9	837	39.9	.118
Sex							
Male	4791	46.7	3749	47.1	1042	45.6	.205
Religion							
Roman Catholic	3643	35.5	3504	44.0	139	6.1	
Protestant	1218	11.9	1163	14.6	55	2.4	
Muslim	36	0.4	31	0.4	5	0.2	
None	1216	11.9	1181	14.8	35	1.5	
Other	66	0.6	65	0.8	1	0.0	
Unknown to the hospice	4075	39.7	2053	25.8	2022	88.7	.004
Living situation							
Alone	4257	53.1	3735	53.4	522	51.2	
With Partner	2803	35.0	2430	34.7	373	36.6	
In Institution	509	6.3	428	6.1	81	7.9	
With Children	435	5.4	393	5.6	42	4.1	
Other	15	0.2	14	0.2	1	0.1	.032
Primary Diagnosis							
Cancer	8345	83.3	6617	84.1	1728	80.6	
Heart Disease	659	6.6	503	6.4	156	7.3	
Pulmonary Disease	217	2.2	159	2.0	58	2.7	
Neurological Disease (inc. CVA)	330	3.3	239	3.0	91	4.2	
Other	466	4.7	354	4.5	112	5.2	<.001

Missing Values: Total n *(Admitted, Not Admitted)* Age 651 *(461, 190),* Living Situation 2235 *(966, 1269)*, Primary Diagnosis 237 *(94, 143)*

Neither age nor sex differed significantly between those admitted and not admitted to inpatient hospice facilities, with most patients in each group aged between 61 and 80 years, with a slightly larger female than male population. Patients who were admitted to inpatient hospice facilities were statistically significant more often lived alone than patients who were not admitted (53.4 and 51.2 %). Though the highest proportion in either group were cancer patients, (84.1 % of admitted patients and 80.6 % of not-admitted patients), the difference between the two groups was statistically significant (Table 1).

### Comparison of diagnosis for inpatient hospice population and national population

**Fig. 1:**
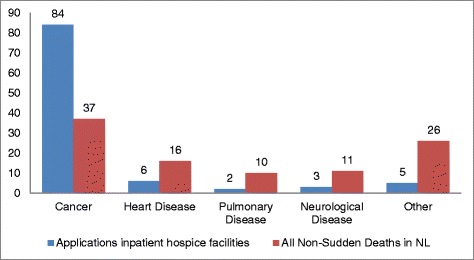
Main diagnosis of those applying for inpatient hospice facility and national figure of cause of death of all people who died unexpectedly and non-suddenly (van der Heide, [10])

The main diagnosis of those applying for inpatient hospice care differed greatly from the spread of causes of non-sudden deaths found nationally. In the studied inpatient hospice group, cancer formed by far the largest population at 84 %, whereas nationally this accounted for 37 % of deaths. The incidence of pulmonary disease as a cause of non-sudden death nationally is five times higher than in the inpatient hospice population, at 10 % against 2 %, and both heart and neurological diseases show an incidence rate that is more than three times higher in the national population than the inpatient hospice population (Fig. 1).

### Application characteristics

**Table 2:** Application Characteristics for inpatient hospice facility

	All applicants (*n* = 10254)		Admitted to inpatient hospice facility (*n* = 7966)		Not admitted to inpatient hospice facility (*n* = 2288)		*P* value
Type of institution	n	%	n	%	n	%	
Standalone hospice facility	3512	34.3	2685	33.7	827	36.1	
PCU in/with Nursing Home	3322	32.4	2514	31.6	808	35.3	
Bijna Thuis Huis	3420	33.4	2767	34.7	653	28.6	<.001
Patient applied from							
Hospital	5210	52.4	4077	51.2	1133	57.4	
Home	3948	39.7	3237	40.6	711	36.0	
Nursing Home	296	3.0	252	3.2	44	2.2	
Care Home	287	2.9	231	2.9	56	2.8	
Inpatient hospice	56	0.6	47	0.6	9	0.5	
Other	142	1.5	121	1.5	21	1.1	<.001
Reason for application^a^							
Wish to die in a hospice	6948	70.5	5571	70.0	1377	72.9	.111
Intensive care/support	5144	52.2	4331	54.4	813	43.1	<.001
Relief for caregivers	4081	41.4	3443	43.2	638	33.8	<.001
Pain and symptom control	3933	39.9	3413	42.9	520	27.5	<.001
Time span of intended care							
Until death	9534	96.2	7616	95.6	1918	98.5	
Respite	379	3.8	350	4.4	29	1.5	<.001
Desired timing							
ASAP	5379	52.5	4413	55.4	966	42.2	
Within 1 month	4210	41.1	3352	44.6	658	28.8	
Just-in-case	665	6.5	1	0	664	29.0	<.001
Who referred							
GP	3021	29.5	2577	32.3	444	19.4	
Nurse (hospital)	3872	37.8	3034	38.1	838	36.6	
Patient or Family	1104	10.8	762	9.6	342	15.0	
Nursing home staff	947	9.2	805	10.1	142	6.2	
District nurse	466	4.5	419	5.3	47	2.1	
Care mediation service	154	1.5	151	1.9	3	0.1	
Other	690	6.7	218	2.7	472	20.6	<.001

Missing Values: Total n *(Admitted, Not Admitted)* Admitted From 315 *(1, 314),* Reason for Application 404 *(4, 400)*, Timespan of Intended Care 341 *(0, 341)*

^a^More than one answer possible

About half of all applicants (52.4 %) were staying in hospital at the time of admission. The most frequent reason for application was the wish to die in a hospice (70.5 %), followed by needing intensive care or support (52.2 %), relieving the caregivers (41.4 %) and needing pain and symptom control (39.9 %). Intensive care and support includes medical, psychological, spiritual and psychosocial aspects of care. About 9 out of 10 applicants wanted to stay in a hospice until death (versus respite care) (96.2 %), and about half of all applicants wanted this care as soon as possible (52.5 %). A statement of a wish to die in a hospice was recorded as a motivation for applying to an inpatient hospice facility, whilst wanting hospice care until death (versus respite care) was recorded as the intended time span of care (Table 2).

There were widespread differences between those who were admitted and those not admitted when looking at application characteristics. A higher percentage of patients who were not admitted were already residing in hospital (57.4 % against 51.2 %). While there was no difference between the two groups in wishing to die in a hospice as reason for application, all three other reasons occurred most often in the group that was admitted to inpatient hospice facilities. Over four-times as many patients admitted for respite were admitted into inpatient hospice facilities than not (4.4 % against 1.5 %). In the group admitted to inpatient hospice facilities, the percentage of patients wanting to be admitted as-soon-as-possible was higher than in the group that was not admitted (55.4 % versus 42.2 %). Finally, there were differences between the two groups in terms of who had referred the patient to inpatient hospice facilities. The percentage of referrals by GPs was larger in the admitted group than in the not admitted group (32.3 % versus 19.4 %) and similarly, the percentage of referrals by patient or family were smaller amongst those admitted (9.6 % versus 15.0 %) (Table 2).

### Associations affecting admittance

**Table 3:** Demographic and application characteristics associated with being admitted for care in inpatient hospice facility

	Univariate^a^		Multivariate^a^	
	OR	95 % CI	OR	95 % CI
Demographic Characteristics				
Age:				
< 60 years	1.05	0.90–1.23		
61–68 years	1.10	0.98–1.21		
81+ years	1.00			
Male (vs female)	1.06	0.97–1.17		
Living alone (vs not)	1.73	1.54–1.95	1.68	1.46–1.94
Main diagnosis:				
-Cancer	1.33	1.13–1.56	1.40	1.11–1.76
-Heart failure	1.15	0.90–1.47	1.27	0.89–1.80
-Lung disease	0.95	0.68–1.33	0.84	0.53–1.34
-Other	1.00		1.00	
Application Characteristics				
Type of inpatient hospice facility:				
-Standalone hospice facility	1.00		1.00	
-‘bijna-thuis-huis’	0.96	0.86–1.07	1.21	1.03–1.43
-PCU in nursing home	1.31	1.16–1.47	1.93	1.60–2.32
Applied from:			^b^	
-Hospital	1.00			
-Home	1.27	1.14–1.41		
-Other	1.39	1.14–1.70		
Reasons for application				
-Wish to die in a hospice (vs not)	0.87	0.77–0.97	0.85	0.72–1.00
-Intensive care/support (vs not)	1.58	1.43–1.75	^b^	
-Relief for caregivers (vs not)	1.49	1.34–1.66	1.18	1.01–1.38
-Pain/symptom control (vs not)	1.97	1.77–2.20	1.72	1.46–2.03
Wanting care until death (vs respite)	3.04	2.07–4.45	3.59	2.11–6.10
Desired timing admission ASAP (vs rest)	1.70	1.55–1.87	1.64	1.42–1.88
Applicant from primary care (vs rest)	1.69	1.51–1.90	1.36	1.17–1.59

^a^univariate and backwards multivariate logistic regression; reference group not being admitted to a hospice

^b^were entered but in the multivariate logistic regression, but did not remain in the analysis until the final step

In the multivariate regression analyses two demographic characteristics were positively associated with being admitted to inpatient hospice facilities: living alone (OR 1.68, 95 % CI 1.46–1.94) and having cancer (OR 1.40, 95 % CI 1.11–1.76). Apart from where the patient would be admitted from and needing intensive support as reason for application, all studied application characteristics remained significant in the analysis. Of different organisational models, PCUs embedded in nursing homes more often admitted patients to care than inpatient hospice facilities (OR 1.93, 95 % CI 1.60–2.32). Of the reasons for application, wishing to die in a hospice was negatively associated with being admitted to inpatient hospice facilities (OR 0.85, 95 % CI 0.72–1.00), though this is not statistically significant. Relieving caregivers (OR 1.18, 95 % CI 1.01–1.38) and needing pain and symptom control (OR 1.72, 95 % CI 1.46–2.03) were positively associated with being admitted to inpatient hospice facilities. Finally, wanting care until death (versus respite care) (OR 3.59, 95 % CI 2.11–6.10), wanting to be admitted as soon as possible (OR 1.64, 95 % CI 1.42–1.88), and being referred by a professional working in primary care (OR 1.36, 95 % CI 1.17–1.59) were positively associated with being admitted to inpatient hospice facilities (Table 3).

### Reason for non-admittance to a hospice

**Fig. 2:**
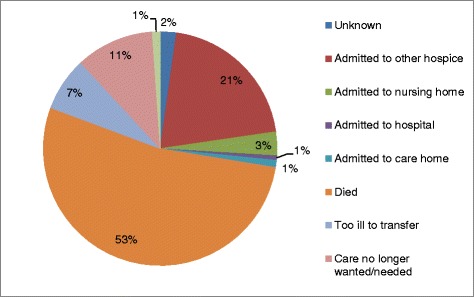
Reason for non-admittance to inpatient hospice facility

The majority of patients who were not admitted after application were not admitted because they had died in between application and the opportunity for admittance (53 %). A further 7 % were too ill to transfer to another care setting, and 11 % stated that the care was no longer wanted or needed. In total, 26 % of patients were transferred to another care setting – be that another inpatient hospice facility, a nursing home, a care home or a hospital. Of these transfers, admission to another inpatient hospice facility was the most common at 21 % (Fig. 2).

## Discussion

### Summary

The data show that certain demographic characteristics differed significantly between patients who were admitted and who were not following application. Primary diagnosis as a characteristic differed between the population who applied for inpatient hospice care and the national records of non-sudden deaths, with more cancer patients applying for inpatient hospice care. Logistic regression showed that patients with a cancer diagnosis were more likely to be admitted to inpatient hospice care.

Application characteristics showed several significant differences between those who were admitted and not. Notably, in regards to where a patient resided at the time of admission, patients were admitted from home rather than hospital. This was reflected in who made the application, with an applicant from a primary care setting being positively associated with the patient being admitted to inpatient hospice facilities. The reason for application was also significant, with a need for pain and symptom control being strongly associated with a patient’s admittance to inpatient hospice care. Timing of care - both in terms of whether patients were seeking care until death or for respite, and how soon they wanted inpatient hospice care to commence – differed significantly between patients who were admitted and not, with admitted patients requesting more urgent care and care until death. The reason recorded for patients not being admitted was primarily because they had died between application and opportunity for admittance.

### Strengths and limitations

The study population was gathered from a database representing over 25 % of inpatient hospice facilities operating in the Netherlands. A large number of deaths were included in the study, giving the results statistical power. As data are a record of admission and stay characteristics and was recorded in a standardised manner, this forms a reliable set of data, as the information gathered is part of the patient’s usual process of care.

An inherent limitation of the study is that this data only cover the professional perspective of care – thus allowing us to identify which patients enter inpatient hospice facilities and why patients might not be admitted, but it does not provide any information on admittance and non-admittance from the patient perspective and the ramifications of these decisions on an experiential level.

### Applications

Hospice care was originally developed to care for the dying cancer population [5] and this is still reflected today. Results showed that a much higher proportion of patients who applied to inpatient hospice services had a primary diagnosis of cancer, when compared to the cause of death for all non-sudden deaths nationwide. This reflects what has been shown in previous international literature – a 2014 paper by Klinger et al. [11] found that ~90 % of patients who died in a hospice in Canada, England and Germany had a cancer diagnosis. Our study shows that, although cancer patients have a somewhat higher chance to be admitted than other patients after applying to inpatient hospice facilities, the main reason for the high percentage of cancer patients in inpatient hospice facilities lies in there being more applications for cancer patients than for other patients. The question is whether this is due to cancer patients more frequently needing inpatient hospice care. One argument against this is that previous studies have reported on the symptom burden experienced by patients with a number of non-malignant diagnoses, and have found that symptoms that may have benefitted from specialist palliative care (pain, breathlessness, social and psychological needs) were common across different patient groups [12, 13]. Ostgathe et al. found that non-cancer patients experience more symptoms than cancer patients [14].

This suggests that the lack of utilization of specialist services by such patients is not due to a lack of need for services that provide holistic relief and concentration on quality of life. Field [15] identified the differences in disease trajectories and defining patients as being “in the terminal stage” as key factors that affect patients with non-malignant conditions access to specialist palliative care. While our results may suggest that non-cancer patients represent an under-represented group, it remains unclear which part of the difference in application for inpatient hospice care in cancer and non-cancer patients is due to under serving, and which part is due to different needs and further literature is needed to address this. The under-representation of non-cancer patients could also be a result of most people assuming that inpatient hospice facilities are primarily for patients with terminal cancer. Earlier it was found in the US that physicians were not good in identifying appropriate candidate diagnosis for inpatient hospice referral [8]. It is also possible that non-cancer patients are under-represented in inpatient hospice facilities as they are more often cared for in nursing homes until death, and are unlikely to transfer to inpatient hospice facilities before death.

### Admittances

Results showed that those admitted to inpatient hospice facilities had more often stated needs for intensive care and support, relief for caregivers and pain and symptom control than applicants that were not admitted. This is concurrent with the WHO definition of palliative care as focusing on “treatment of pain and other problems, physical, psychosocial and spiritual” [16]. Looking at the application characteristics that increase the chance of being admitted, it seems that certain characteristics might be related to a higher need for inpatient hospice care: patients living alone, needing pain relief and wanting care as soon as possible and until death.

Little over half of the admittances followed a hospital admission – this may be an indicator that earlier intervention, resulting in more care needs, then precludes returning home for patients. That patients who applied from hospital were somewhat less frequently admitted to a hospice is likely to be due to dying before admission. Finally, the result that the most frequent reason for not being admitted to inpatient hospice facilities is the death of the patient suggests that it may have been beneficial to consider applying for inpatient hospice care earlier on for at least part of this group.

## Conclusions

This study suggests that when applying for inpatient hospice care, patients who seem most urgently in need of hospice care are more frequently admitted, yet our study has several implications for the process of admitting patients to a hospice. Firstly, our results suggest that non-cancer patients are an under-represented group, especially because they are less often referred to hospice care. Previous studies have highlighted palliative needs of non-cancer patients being similar or more. Staff should consider application for non-cancer patients based on need for symptom control and palliation, rather than basing a decision on diagnosis. Secondly, the most frequent reason for not being admitted to inpatient hospice facilities is the death of the patient, suggesting that this group might have benefitted from considering applying to an inpatient hospice facility earlier on – at least in part. Also in this situation considering application based on the need for symptom control and palliation rather than on diagnosis could help. Finally, hospices themselves should recognize stated needs for care, and standalone hospice facilities, PCUs and bijna-thuis-huizen could work together regionally so that places can be found for applicants even if this is not in the original institution of choice.
